# Supplementary material for: Why do people resist AI-based autonomous cars?: Analyzing the impact of the risk perception paradigm and conditional value on public acceptance of autonomous vehicles
Source: PLoS One. 2025 Feb 6;20(2):e0313143. doi: 10.1371/journal.pone.0313143 (PMC11801528; doi:10.1371/journal.pone.0313143)
Supplement: S1 Appendix — (DOCX) [file pone.0313143.s001.docx]

Appendix 1. Fixed Effects Parameter Estimates

|  | | | 95% Confidence Interval | |  | | | |
| --- | --- | --- | --- | --- | --- | --- | --- | --- |
| Names | Estimate | SE | Lower | Upper | β | df | t | p |
| (Intercept) | 1.4192 | 0.04323 | 1.3344 | 1.50395 | 0 | 1791 | 32.8298 | < .001 |
| Gender | -0.6127 | 0.08574 | -0.7809 | -0.44458 | -0.13724 | 1791 | -7.1465 | < .001 |
| Age | -0.0111 | 0.00347 | -0.0179 | -0.0043 | -0.0646 | 1791 | -3.2009 | 0.001 |
| Income | 0.1868 | 0.07003 | 0.0495 | 0.32413 | 0.05118 | 1791 | 2.6675 | 0.008 |
| Education | 0.2355 | 0.09254 | 0.054 | 0.41696 | 0.05011 | 1791 | 2.5446 | 0.011 |
| Risk | -0.1062 | 0.04845 | -0.2012 | -0.01114 | -0.04206 | 1791 | -2.1913 | 0.029 |
| Benefit | 0.6175 | 0.07127 | 0.4777 | 0.75725 | 0.1929 | 1791 | 8.6642 | < .001 |
| Trust | 0.083 | 0.07333 | -0.0608 | 0.22684 | 0.02678 | 1791 | 1.1323 | 0.258 |
| Knowledge | -0.0131 | 0.05646 | -0.1238 | 0.09763 | -0.00501 | 1791 | -0.2322 | 0.816 |
| Image | 0.9448 | 0.07729 | 0.7932 | 1.09634 | 0.25891 | 1791 | 12.2236 | < .001 |
| Condition | 0.2421 | 0.01641 | 0.2099 | 0.27429 | 0.29763 | 1791 | 14.7547 | < .001 |
| Risk ✻ Condition | -4.79e−4 | 0.0158 | -0.0315 | 0.03052 | -5.21e−4 | 1791 | -0.0303 | 0.976 |
| Benefit ✻ Condition | -0.0858 | 0.02325 | -0.1313 | -0.04016 | -0.07354 | 1791 | -3.6888 | < .001 |
| Trust ✻ Condition | 0.076 | 0.02269 | 0.0315 | 0.12046 | 0.06726 | 1791 | 3.3479 | < .001 |
| knowledge ✻ condition | -0.0128 | 0.01813 | -0.0484 | 0.02273 | -0.01348 | 1791 | -0.708 | 0.479 |
| Image ✻ Condition | -0.0911 | 0.02563 | -0.1414 | -0.04086 | -0.06855 | 1791 | -3.5558 | < .001 |

Appendix 2. Interaction Effect between Perceived benefit and Conditional value (Dep var: Acceptance of Autonomous vehicles)

| **Moderator levels** |  | |  | | | |
| --- | --- | --- | --- | --- | --- | --- |
| **condition** | **Estimate** | **SE** | **β** | **df** | **t** | **p** |
| Mean-1-SD | 0.853 | 0.0969 | 0.266 | 1791 | 8.8 | < .001 |
| Mean | 0.617 | 0.0713 | 0.193 | 1791 | 8.66 | < .001 |
| Mean+1-SD | 0.382 | 0.0944 | 0.119 | 1791 | 4.05 | < .001 |
| Note. Simple effects are estimated keeping constant other independent variable(s) in the model | | | | | | |

Appendix 3. Interaction Effect between Trust and Conditional Value(Dep var: Acceptance of Autonomous vehicles)

| **Moderator levels** |  | |  | | | |
| --- | --- | --- | --- | --- | --- | --- |
| **condition** | **Estimate** | **SE** | **β** | **df** | **t** | **p** |
| Mean-1-SD | -0.1255 | 0.096 | -0.0405 | 1791 | -1.31 | 0.191 |
| Mean | 0.083 | 0.0733 | 0.0268 | 1791 | 1.13 | 0.258 |
| Mean+1-SD | 0.2915 | 0.0964 | 0.094 | 1791 | 3.02 | 0.003 |
| Note. Simple effects are estimated keeping constant other independent variable(s) in the model | | | | | | |

Appendix 4. Interaction Effect between Image and Conditional Value(Dep var: Acceptance of Autonomous vehicles)

| **Moderator levels** |  | |  | | | |
| --- | --- | --- | --- | --- | --- | --- |
| **condition** | **Estimate** | **SE** | **β** | **df** | **t** | **p** |
| Mean-1-SD | 1.195 | 0.1034 | 0.327 | 1791 | 11.56 | < .001 |
| Mean | 0.945 | 0.0773 | 0.259 | 1791 | 12.22 | < .001 |
| Mean+1-SD | 0.695 | 0.1057 | 0.19 | 1791 | 6.57 | < .001 |
| *Note.* Simple effects are estimated keeping constant other independent variable(s) in the model | | | | | | |
