# Supplementary material for: Why do people resist AI-based autonomous cars?: Analyzing the impact of the risk perception paradigm and conditional value on public acceptance of autonomous vehicles
Source: PLoS One. 2025 Feb 6;20(2):e0313143. doi: 10.1371/journal.pone.0313143 (PMC11801528; doi:10.1371/journal.pone.0313143)
Supplement: Attachment — Submitted filename: Revision Note-version 2.docx [file pone.0313143.s002.docx]

Cover Letter

20 September 2024

Dear Editors:

We submit an revisited manuscript entitled “Why Do People Resist AI-based Autonomous Cars?: Analyzing the Impact of the Risk Perception Paradigm and Conditional Value on Public Acceptance of Autonomous Vehicles” for consideration by PLOS ONE.

Comments from reviewers were very helpful for the development of the paper. We tried to revise all of things that the reviewers pointed out. Reviewers’ comments gave us very valuable insight and direction for revision.

Once again, if viewers give us a good comment, we will use it to improve our paper.

I confirm that this study is original and is our own work. It has not been published elsewhere, nor is it currently under consideration for publication elsewhere. It has been checked by a native English speaker with expertise in social sciences.

Given its research focus and methodology, I reckon that it is more appealing to a scientific audience. Lastly, the authors are available to review at least three new submissions for PLOS ONE within the next 12 months.

I have no conflicts of interest to disclose.

Please address all correspondence concerning this manuscript to me at

seoyongkim@ajou.ac.kr.

Thank you for your consideration of this manuscript.

Sincerely,

Seoyong Kim

Comment from Reviewer 1 and Revision

*Thank you for the kind reviews. What comments from reviewer was very helpful for the development of the paper. We tried to revise all of things that the reviewer pointed out. Once again, if reviewer gives us a good comment, we will use it to improve our paper.*

*Best regards,*

*Authors*

□ Reviewer #1

□ Comment 1: The abstract mentions the use of the "risk perception paradigm and conditional value" but does not clarify how these theoretical models were specifically applied in the analysis. Providing more detail on how these frameworks were operationalized would strengthen the abstract's clarity and rigor.

○ Answer: The reason for choosing 11 values was discussed in detail as follows and added to the text.

○ Revision: We explained how we analyzed the theoretical model of this study in the abstract.

*We set up the variable from risk perception paradigm as independent variable and the conditional value as moderating variable in explaining the acceptance of AI-based autonomous vehicles. For this work, the analysis was conducted in two stages. Initially, a regression analysis was performed to determine the impact of the risk perception paradigm and conditional value on the acceptance of autonomous vehicles. Secondly, a moderation analysis was conducted to determine whether the perception of self-driving taxis moderates the relationship between the risk perception paradigm and the acceptance of autonomous vehicle.*

*The study revealed that the acceptance of autonomous vehicles is influenced by a number of factors, including knowledge, image, conditional value, and perceived risks. Additionally, the relationship with perceived benefits, image and autonomous vehicle is moderated by conditional value.*

□ Comment 2: The abstract lacks information on the methodology used to explore the causal relationships, such as the sample size, data collection methods, or statistical techniques employed. Including these details would provide better insight into the robustness and validity of the findings.

○ Answer: We have briefly added the research design section to the abstract.

○ Revision:

*The survey was conducted between July 8 and 17, 2019. In order to increase the representativeness of the sample, a quota sampling method was adopted, based on considering gender and region. The sample size of this survey is 2,000 people. The maximum allowable sampling error is ±2.2 percentage points at the 95% confidence level, assuming random sampling. The survey method was a web survey. The URL was sent via mobile phone text and email, and then respondents answered a self-completion survey. According to the response statistics, 26,231 people requested the survey, 3,973 people participated in the survey, and 2,000 people completed the survey. The participation rate is 7.6% of requests. We adopted regression and moderation analysis as main statistical analysis methods.*

□ Comment 3: The introduction should clearly conclude with a distinct section highlighting the novel contributions of your work.

○ Answer: We briefly add theory to the introduction to highlight what can be gained from this study.

○ Revision:

*The objective of this study is to identify the variables that influence the acceptance of autonomous vehicles at both the psychological and conditional levels. In order to examine the psychological dimension, we employ the risk perception paradigm, which allows us to capture an individual's subjective perception of risk. The risk perception paradigm, as proposed by Slovic et al. (1981), asserts that the subjective perception of risk is dependent on the individual in question. Consequently, the acceptance of a risky object is influenced by the subjective perception of risk. The specific variables related to the risk perception paradigm include perceived risk, perceived benefit, trust, knowledge, and image. In addition, this study proposes a conditional value variable based on the theory of consumption value. The theory of consumption value is proposed by Sheth, Newman, & Gross (1991) that consumers decide whether to purchase a product or not and whether to use the product or not. The conditional value of this study is used to check whether there will be a change in attitude toward the acceptance of autonomous vehicles if one assumes that one is a taxi driver. Specifically, it was conducted to check whether there is a causal relationship with the acceptance of autonomous vehicles and whether it plays a moderating role in the causal relationship between the risk perception paradigm and the acceptance of autonomous vehicles. The objective of this study is to provide policy implications for the future acceptance of autonomous vehicles and suggestions for their future development.*

*Such a theoretical approach can contribute to research in this area in the following ways; The model proposed in this study will contribute to the development of future AI-based acceptance models, as there have been few theoretical models what explains the acceptance of AI-based cars.*

□ Comment 4: The literature review could benefit from more explorations of previous studies. This will provide a richer context and demonstrate how your work builds upon or diverges from established research.

○ Answer: We added literature review supplemented: Summary of autonomous vehicle research, limitations of previous studies, and theoretical contribution of knowledge and trust.

○ Revision:

*(Page 5)Hewitt et al. (2019) introduced the 'Autonomous Vehicle Acceptance Model,' which integrates the Unified Theory of Acceptance and Use of Technology (UTAUT), the Technology Acceptance Model (TAM), and the TAM2 model, drawing from various frameworks related to technology acceptance. Similarly, Nastjuk (2020) sought to explain the intention to accept autonomous vehicles by incorporating individual-level variables and systemic characteristics into the TAM framework. In a study by Mara and Meyer (2022), variables used in published research on autonomous vehicle acceptance were categorized into three main groups: user-specific determinants (e.g., demographic and personality characteristics), vehicle-specific determinants (e.g., perceived safety, predictability, and appearance), and situational determinants (e.g., road conditions).*

*Despite the active research on autonomous vehicle acceptance, particularly in light of the anticipated development of these technologies, existing studies face several limitations. First, much of the research is driven by specific variables rather than comprehensive theoretical models, which may lead to bias in the selection of variables. Second, although autonomous vehicles could be perceived as a threat due to social concerns such as safety issues, ethical dilemmas, and job displacement, these aspects have not been adequately explored. The introduction of new technologies often brings the potential for new risks, and Mara and Meyer (2022) highlighted that psychological factors are among the key variables influencing the acceptance of autonomous vehicles. In response to these gaps, this study proposes a theoretical model grounded in the risk perception paradigm, examining the acceptance of autonomous vehicles while considering conditional value.*

*(Page 9)The first stage of the process of adopting innovations by Rogers (1983) is described as knowledge. Knowledge here includes the degree of exposure to innovations, such as technical knowledge of the innovation technology, experience with the innovation technology, and information acquisition (Rogers, 1983).*

*(Page 9)Image plays a crucial role in technology acceptance research. In his innovation diffusion theory, Rogers (1983) emphasized that during the Persuasion stage of technology acceptance, the key factor is the positive or negative perception or attitude toward the technology. Building on this idea, Davis (1985) incorporated ‘attitude’ as a significant factor in his technology acceptance model. Here, attitude refers to the positive or negative perception of the technology, which can be largely influenced by the image of the technology. The image, therefore, becomes a critical determinant in shaping users' perceptions and, ultimately, their acceptance of new technologies.*

+ Hewitt, C., Theocharis, A., Advait, S & Ioannis, P. Assessing Public Perception of Self-Driving Cars: the Autonomous Vehicle Acceptance Model. 2019. 10.1145/3301275.3302268.

+ Mara, M., Meyer, K. Acceptance of Autonomous Vehicles: An Overview of User-Specific, Car-Specific and Contextual Determinants. In: Riener, A., Jeon, M., Alvarez, I. (eds) User Experience Design in the Era of Automated Driving. Studies in Computational Intelligence, vol 980. Springer, Cham: 2022. <https://doi.org/10.1007/978-3-030-77726-5_3>

+ Nastjuk, I., Herrenkind, B., Marrone, M., Brendel, A, B., Kolbe, L, M. What drives the acceptance of autonomous driving? An investigation of acceptance factors from an end-user's perspective, Technological Forecasting and Social Change, 2020; Volume 161. <https://doi.org/10.1016/j.techfore.2020.120319>.

+ Othman, K. Public acceptance and perception of autonomous vehicles: a comprehensive review. AI Ethics. 2021; 1, 355–387. https://doi.org/10.1007/s43681-021-00041-8.

+ Rogers. Diffusion of Innovations. 1983. New York: Free Press. (Original work published 1962).

□ Comment 5: The discussion section needs to be expanded to more thoroughly analyze the results.

○ Answer: We have added some of the discussion-worthy content from the analysis results.

○ Revision:

*Based on the analysis, five hypotheses were formulated regarding the influence of the risk perception paradigm and contingent value on the acceptance of autonomous vehicles. Within the risk perception paradigm, perceived risk was found to negatively affect the acceptance of autonomous vehicles (Hypothesis 1). Conversely, perceived benefits, trust, and image were found to have a positive effect on the acceptance of robotic cars (Hypotheses 2, 3, and 5). These results indicate that positive perceptions of AI technology significantly contribute to the acceptance of autonomous vehicles, suggesting the need for strategies to reduce perceived risks and negative perceptions, while simultaneously enhancing trust and positive perceptions of AI technology itself to increase public acceptance.*

*Hypothesis 6 was also supported, as respondents with a more optimistic view of autonomous vehicle adoption were more likely to approve of their use, as measured by a conditional value variable, which asked whether respondents would support autonomous vehicle adoption if they worked as taxi drivers. This outcome can be interpreted in light of the fact that, for taxi drivers, the introduction of autonomous vehicles may reduce their technical role, thereby lowering their workload. Furthermore, the conditional value variable partially supported Hypothesis 7 by moderating the relationship between perceived benefits, image, and acceptance of robotic cars within the risk perception paradigm.*

*The analytical outcomes varied depending on the inclusion of demographic variables as control factors. In Model 1, where control variables were excluded, greater knowledge of AI technologies positively influenced the acceptance of autonomous vehicles, while trust had no significant effect. However, in Model 2, which incorporated demographic control variables, the positive influence of knowledge on acceptance became insignificant, while trust became both positive and significant. Given that autonomous vehicles are part of the Fourth Industrial Revolution, knowledge of AI-related technologies is likely to be higher among individuals with higher levels of education. Therefore, it is plausible that the significance of the knowledge variable was attenuated by the inclusion of control variables such as income and education, which are likely correlated with knowledge levels. Similarly, the insignificance of the trust variable in Model 1, which became significant in Model 2, can be explained by the general increase in trust that accompanies specialized knowledge in a particular field. In Model 1, the effect of knowledge may have overshadowed trust, but in Model 2, the influence of knowledge was controlled, allowing trust to emerge as a significant factor in the acceptance of autonomous vehicles.*

*This study contributes to the existing body of literature by examining the impact of psychological factors on the acceptance of autonomous vehicles. Whereas prior research has largely focused on demographic, economic, and technological dimensions, this study incorporates control variables from previous studies to assess the role of psychological factors in shaping public acceptance.*

□ Comment 6: Zero visualization efforts.

○ Answer: We changed the figure and added to contribution of our findings in conclusion. Please, give us tip for Zero visualization methods.

□ Comment 7: I can not help but notice the name of such figures, as 3, are not academic. Please revise the entire manuscript for such similarity.

○ Answer: Based on extensive theoretical review, Robotic cars were changed to Autonomous Vehicles, and variable names were modified overall.

○ Revision:

□ Comment 8: The first paragraph of the conclusion should succinctly summarize the contributions of the study in past tense, clearly stating what has been accomplished and the impact it has on the field.

○ Answer: We did the first paragraph of the conclusion summarizes the research results and describes the differences from previous studies.

○ Revision:

*This study employed regression analysis and moderation analysis, incorporating conditional value variables related to the risk perception paradigm and situational conditions that may influence the adoption of autonomous vehicles, with the aim of identifying psychological factors that affect their acceptance. The analysis revealed that within the risk perception paradigm, perceived risk exerted a negative influence on the acceptance of autonomous vehicles, whereas perceived benefits, trust, and image had a positive influence. Furthermore, conditional value grounded in bounded rationality demonstrated a positive effect on the acceptance of autonomous vehicles and moderated the relationship between perceived benefits, image, and their acceptance.*

*This study differs from previous research by attempting to explain the acceptance of autonomous vehicles from a psychological perspective, particularly focusing on individual risk perception, rather than solely relying on the technology acceptance model, which has been the primary focus of earlier studies.*

□ Comment 9: The second paragraph of the conclusion should provide clear and actionable future recommendations. This will guide subsequent research and highlight potential areas for further investigation based on your findings.

○ Answer: We have added the following future recommendations

○ Revision:

*Second, perceived benefits, image, and trust were found to have a strong positive effect on the acceptance of autonomous vehicles. This finding underscores the importance of fostering positive psychological dimensions in enhancing the acceptance of autonomous vehicles. Therefore, promoting the benefits and positive image associated with autonomous vehicles is essential. However, the crux of fostering a positive perception lies in whether autonomous vehicles can adequately secure reliability at a technical level. To enhance reliability, it is imperative to address and eliminate risk factors, both direct and indirect, such as improving the safety of autonomous vehicles, reducing the likelihood of accidents, and resolving ethical dilemmas.*

*Third, the study observed that conditional value had a significant positive effect on the acceptance of autonomous vehicles. This suggests that a favorable attitude toward autonomous vehicles, particularly when considered from the perspective of taxi drivers, can strengthen their acceptance. This result can be interpreted in two ways. First, it may indicate that at the time of the study, there was a high level of trust in autonomous vehicles, which positively influenced their acceptance. Second, it suggests that the expected reduction in taxi drivers' workload due to autonomous vehicles contributed to this positive effect. It is widely acknowledged that the rise of AI technology poses a threat to the continuity and stability of workers' jobs (Arslan et al., 2021; Rampersad, 2020). Taxi drivers, in particular, are often highlighted as a profession at high risk of job replacement by autonomous vehicles. However, it is also anticipated that the advancement of AI technology will not necessarily result in job displacement but could instead lead to a coexistence of AI technology and human workers (Jaiswal et al., 2021; Wilson & Daugherty, 2019). In reality, the complete replacement of jobs by AI technology remains politically, socially, and economically uncertain (Willcocks, 2020). Moreover, AI technology has never operated entirely independently without human oversight (Chuang & Graham, 2018), and even if it were to do so, it would still face unavoidable ethical dilemmas. These ethical dilemmas extend beyond job replacement to encompass all potential ethical issues that may arise from the deployment of AI technology. This study confirms that there is an expectation among people for the coexistence of workers and AI technology, rather than the outright replacement of jobs through AI advancements. This finding suggests a potential direction for the development of AI technology, including autonomous vehicles, where human-AI collaboration is emphasized.*

+ Arslan, C. Cooper, Z. Khan, I. Golgeci, I. Ali. Artificial intelligence and human workers interaction at team level: a conceptual assessment of the challenges and potential HRM strategies Int. J. Manpow. 2021; 10.1108/IJM-01-2021-0052.

+ Chuang, C.M. Graham Embracing the sobering reality of technological influences on jobs, employment and human resource development: a systematic literature review Eur. J. Training Dev., 42 (7/8) 2018; pp. 400-416.

+ Jaiswal, C.J. Arun, A. Varma Rebooting employees: upskilling for artificial intelligence in multinational corporations Int. J. Hum. Resour. Manag. 2021; pp. 1-30, 10.1080/09585192.2021.1891114

+ Rampersad, G. Robot will take your job: innovation for an era of artificial intelligence J. Bus. Res., 2020; 116. pp. 68-74, 10.1016/j.jbusres.2020.05.019

+ Willcocks, L. Robo-Apocalypse cancelled? Reframing the automation and future of work debate J. Inf. Technol. 2020; 35 (4) pp. 286-302, 10.1177/0268396220925830

+ Wilson, H, J., P.R. Daugherty Creating the symbiotic AI workforce of the future MIT Sloan Manag. Rev. 2019; 61:(1), pp. 1-4 https://sloanreview.mit.edu/article/creating-the-symbiotic-ai-workforce-of-the-future/

*-We are very grateful for the comment reviewer gave. If reviewer gives us additional comments, we will revise it again. Thanks again for very good comment-*

Comment from Reviewer 2 and Revision

*Dear reviewer*

*Thank you for the kind reviews. What comments from reviewer was very helpful for the development of the paper. We tried to revise all of things that the reviewer pointed out. Once again, if reviewer gives us a good comment, we will use it to improve our paper.*

*Best regards,*

*Authors*

□ Reviewer #2: The article provides a timely analysis of the factors that influence public acceptance of autonomous vehicles, focusing on risk perception and conditional value. The study is grounded in a solid theoretical framework and offers valuable insights into the psychological and contextual factors that shape public attitudes toward the self-driving car technology:

□ Comment 1: The development of the hypotheses could be more clearly linked to the theoretical framework, particularly in explaining how knowledge influences risk perception.

○ Answer: We added the literature review for clearing the link between hypotheses and theoretical framework. Also, added how knowledge influence risk perception.

○ Revision:

* Knowledge

The first stage of the process of adopting innovations by Rogers (1983) is described as knowledge. Knowledge here includes the degree of exposure to innovations, such as technical knowledge of the innovation technology, experience with the innovation technology, and information acquisition (Rogers, 1983). The greater one's knowledge of a technology, the more accurately they can assess it. A deeper understanding allows for a more nuanced evaluation of both minor risks and significant benefits.

In empirical study, Nastjuk et al. (2020) showed the determinants of user acceptance of autonomous driving; the social and individual factors, as well as system characteristics determine it. In particular, knowledge such as relative advantage, compatibility, enjoyment and price valuation about autonomous driving significantly influence the acceptance of autonomous deriving. Ward et al. (2017) demonstrated that increasing knowledge, such as informational materials may enhance positive feelings about the technology. Such knowledge can increase people’s benefit perceptions. Younger generations thought that safety features in new vehicles were more important than older generations; they had greater knowledge of automated vehicles, which was positively correlated with trust in the technology. However, knowledge has not always the positive impact on acceptance. If people get more knowledge about negative contents toward specific automated vehicles, they will naturally show more opposing position about them. Othman, K. (2023) demonstrated a negative change in public attitudes towards AVs as knowledge levels increased. When there is a 1% increase in knowledge, it corresponded to a 0.65% decrease in interest, a 0.68% decline in trust

*Theoretical framework

Hewitt et al. (2019) introduced the 'Autonomous Vehicle Acceptance Model,' which integrates the Unified Theory of Acceptance and Use of Technology (UTAUT), the Technology Acceptance Model (TAM), and the TAM2 model, drawing from various frameworks related to technology acceptance. Similarly, Nastjuk (2020) sought to explain the intention to accept autonomous vehicles by incorporating individual-level variables and systemic characteristics into the TAM framework. In a study by Mara and Meyer (2022), variables used in published research on autonomous vehicle acceptance were categorized into three main groups: user-specific determinants (e.g., demographic and personality characteristics), vehicle-specific determinants (e.g., perceived safety, predictability, and appearance), and situational determinants (e.g., road conditions). Despite the active research on autonomous vehicle acceptance, particularly in light of the anticipated development of these technologies, existing studies face several limitations. First, much of the research is driven by specific variables rather than comprehensive theoretical models, which may lead to bias in the selection of variables. Second, although autonomous vehicles could be perceived as a threat due to social concerns such as safety issues, ethical dilemmas, and job displacement, these aspects have not been adequately explored. The introduction of new technologies often brings the potential for new risks, and Mara and Meyer (2022) highlighted that psychological factors are among the key variables influencing the acceptance of autonomous vehicles. In response to these gaps, this study proposes a theoretical model grounded in the risk perception paradigm, examining the acceptance of autonomous vehicles while considering conditional value.

+ Evans, G., & Durant, J. The relationship between knowledge and attitudes in the public understanding of science in Britain. Public Understanding of Science, 1995; 4(1), 57.

+ Hewitt, C., Theocharis, A., Advait, S & Ioannis, P. Assessing Public Perception of Self-Driving Cars: the Autonomous Vehicle Acceptance Model. 2019. 10.1145/3301275.3302268.

+ Mara, M., Meyer, K. Acceptance of Autonomous Vehicles: An Overview of User-Specific, Car-Specific and Contextual Determinants. In: Riener, A., Jeon, M., Alvarez, I. (eds) User Experience Design in the Era of Automated Driving. Studies in Computational Intelligence, vol 980. Springer, Cham: 2022. <https://doi.org/10.1007/978-3-030-77726-5_3>

+ Nastjuk, I., Herrenkind, B., Marrone, M., Brendel, A, B., Kolbe, L, M. What drives the acceptance of autonomous driving? An investigation of acceptance factors from an end-user's perspective, Technological Forecasting and Social Change, 2020; Volume 161. <https://doi.org/10.1016/j.techfore.2020.120319>.

+ Othman, K. Public acceptance and perception of autonomous vehicles: a comprehensive review. AI Ethics. 2021; 1, 355–387. https://doi.org/10.1007/s43681-021-00041-8.

+ Othman, K. Exploring the evolution of public acceptance towards autonomous vehicles with the level of knowledge. Innovative Infrastructure Solutions, 2023; 8(8), 208.

+ Ward, C., Raue, M., Lee, C., D’Ambrosio, L., & Coughlin, J. F. Acceptance of automated driving across generations: The role of risk and benefit perception, knowledge, and trust. In Human-Computer Interaction. User Interface Design, Development and Multimodality: 19th International Conference, HCI International 2017, Vancouver, BC, Canada, July 9-14, 2017, Proceedings, Part I 19 (pp. 254-266). 2017; Springer International Publishing.

□ Comment 2: The moderation analysis can benefit from a more detailed discussion of the results and their implications, especially regarding different demographic groups.

○ Answer: We added the detailed discussion of the result and their implication

○ Revision:

*Fourth, the conditional value variable had a moderating effect on the relationship between perceived benefits and image and acceptance of AI-based autonomous vehicles. We set up conditional question ‘if you were a taxi driver, would you favor the introduction of self-driving cars?’ A higher value indicates a higher level of favor toward AI-based autonomous vehicles. Taxi drivers are likely to be the most affected by the widespread adoption of autonomous vehicles. Therefore, conditional effect implies that a positive response to this question suggests that the respondent perceives the risks associated with autonomous vehicles to be minimal.*

*Increased perceived benefits and positive emotions are associated with greater acceptance of AI-based autonomous vehicles, but this effect is conditional. The positive impact of perceived benefits and positive emotions is the case when there is a higher condition effect. This means that even if the benefits and image of autonomous vehicles are good, acceptance of AI-based autonomous vehicles is higher when there is less damage to the situation involved.*

*The implications of these findings are that, first, in order to increase the acceptance of AI-based autonomous vehicles, it is necessary to pursue ways to minimize industrial damage at the structural level, not only to increase utility. Second, the acceptance issue is not just an individual choice problem, but also a social group choice problem, which means that it is necessary to consider the class of workers who are affected by the development of science and technology. Third, the influence of conditional variables is greater in situations where perceived benefits and positive emotions are low. This suggests that in the negative context of AI-based autonomous vehicles, more consideration should be given to social side effects and harms, especially in the context of negative perceptions of AI-based autonomous vehicles.*

□ Comment 3: The study’s reliance on cross-sectional data limits the ability to draw causal inferences. While this limitation is acknowledged, the discussion would be enhanced by suggesting how future research could address this issue, perhaps through longitudinal studies.

○ Answer: The limitations of the study and future research were added as follows: (1) limitations of cross-sectional study and need for longitudinal study, (2) clear design of contingent value variables, and (3) diversification of risks that may affect acceptance.

○ Revision:

*This study examined variables influencing the acceptance of autonomous vehicles, anticipated through advancements in AI technology. However, several limitations should be noted. First, the study is susceptible to common source bias, which arises from using a single survey dataset to measure variables. This approach limits the ability to capture changes over time or in response to policy shifts, as cross-sectional data collected at a single point in time was employed. To address this limitation, future research should consider longitudinal studies that can track changes in AI technology risk perception and the evolving acceptance of autonomous vehicles over time.*

*Second, the conditional value variables in this study require clearer design. If the potential for job loss were explicitly integrated into the items used for conditional variables, respondents' decision-making based on bounded rationality would likely be more evident. Future research should clearly present and incorporate factors that may be perceived as individual threats, such as job loss, from a social and ethical perspective.*

*Third, this study demonstrated that subjective risk perception influences the acceptance of autonomous vehicles. However, future studies should also aim to measure the perception of actual risks that are expected to materialize. By identifying which risks do or do not impact acceptance, future research can contribute to improving acceptance through comprehensive risk management strategies.*

□ Comment 4: The discussion on the ethical implications of autonomous vehicles could be expanded to include considerations beyond job displacement, such as data privacy and social inequality.

○ Answer: The contents of the questionnaire were presented in a table, and the response scale and reliability were indicated and added as follows. We added the explanation about the questionnaire design structure in text.

○ Revision: *Since this study is about the acceptance of autonomous vehicles, we were unable to add discussions on data privacy and social inequality, but we briefly try to describe the ethical dilemma. In order to resolve the ethical dilemma, we described the coexistence of AI technology and humans.*

*It is widely acknowledged that the rise of AI technology poses a threat to the continuity and stability of workers' jobs (Arslan et al., 2021; Rampersad, 2020). Taxi drivers, in particular, are often highlighted as a profession at high risk of job replacement by autonomous vehicles. However, it is also anticipated that the advancement of AI technology will not necessarily result in job displacement but could instead lead to a coexistence of AI technology and human workers (Jaiswal et al., 2021; Wilson & Daugherty, 2019). In reality, the complete replacement of jobs by AI technology remains politically, socially, and economically uncertain (Willcocks, 2020). Moreover, AI technology has never operated entirely independently without human oversight (Chuang & Graham, 2018), and even if it were to do so, it would still face unavoidable ethical dilemmas. These ethical dilemmas extend beyond job replacement to encompass all potential ethical issues that may arise from the deployment of AI technology. This study confirms that there is an expectation among people for the coexistence of workers and AI technology, rather than the outright replacement of jobs through AI advancements. This finding suggests a potential direction for the development of AI technology, including autonomous vehicles, where human-AI collaboration is emphasized.*

+ Arslan, C. Cooper, Z. Khan, I. Golgeci, I. Ali. Artificial intelligence and human workers interaction at team level: a conceptual assessment of the challenges and potential HRM strategies Int. J. Manpow. 2021; 10.1108/IJM-01-2021-0052.

+ Chuang, C.M. Graham Embracing the sobering reality of technological influences on jobs, employment and human resource development: a systematic literature review Eur. J. Training Dev., 42 (7/8) 2018; pp. 400-416.

+ Jaiswal, C.J. Arun, A. Varma Rebooting employees: upskilling for artificial intelligence in multinational corporations Int. J. Hum. Resour. Manag. 2021; pp. 1-30, 10.1080/09585192.2021.1891114

+ Rampersad, G. Robot will take your job: innovation for an era of artificial intelligence J. Bus. Res., 2020; 116. pp. 68-74, 10.1016/j.jbusres.2020.05.019

+ Willcocks, L. Robo-Apocalypse cancelled? Reframing the automation and future of work debate J. Inf. Technol. 2020; 35 (4) pp. 286-302, 10.1177/0268396220925830

+ Wilson, H, J., P.R. Daugherty Creating the symbiotic AI workforce of the future MIT Sloan Manag. Rev. 2019; 61:(1), pp. 1-4 https://sloanreview.mit.edu/article/creating-the-symbiotic-ai-workforce-of-the-future/

□ Comment 5:  In statistical analysis, providing effect sizes alongside p-values would also help readers understand the practical significance of the findings.

○ Answer: Details of the questionnaire collection process were additionally explained in the text as follows.

○ Revision: We provide the effect size.

To see the effect size, we calculated cohen's d and the results are shown in Table 2. Except for gender and education, the groups of independent variables were categorized into low group for those below the mean value and high group for those above the mean value. Education was categorized into high school or less and college or higher. The effect size is categorized as small effect size up to .3, medium effect size up to .7, and large effect size up to 1. When looking at the overall effect size, education and income have relatively small values, gender, age, risk, trust, knowledge, and conditions have medium values, and benefits, and positive image have relatively large values.

**Tab. 2. Anova-test and Effect Size**

|  | Group | Mean | S.D. | T-value | P-value | Cohen'd | Effect-size r |
| --- | --- | --- | --- | --- | --- | --- | --- |
| Gender | Male | 1.80 | 2.22 | 105.64 | 0.00 | 0.46 | 0.23 |
|  | Female | 0.80 | 2.10 |  |  |  |  |
| Age | Low | 0.00 | 2.64 | 3.37 | 0.07 | -0.53 | -0.26 |
|  | High | 1.29 | 2.21 |  |  |  |  |
| Education | Low | 0.94 | 2.13 | 25.55 | 0.00 | -0.24 | -0.12 |
|  | High | 1.46 | 2.24 |  |  |  |  |
| Income | Low | 1.14 | 2.20 | 18.50 | 0.00 | -0.20 | -0.10 |
|  | High | 1.59 | 2.21 |  |  |  |  |
| Risk | Low | 1.76 | 2.23 | 44.96 | 0.00 | 0.32 | 0.16 |
|  | High | 1.05 | 2.17 |  |  |  |  |
| Benefit | Low | 0.49 | 2.18 | 233.74 | 0.00 | -0.69 | -0.32 |
|  | High | 1.93 | 2.02 |  |  |  |  |
| Trust | Low | 0.93 | 2.21 | 100.39 | 0.00 | -0.47 | -0.23 |
|  | High | 1.94 | 2.07 |  |  |  |  |
| Knowledge | Low | 0.92 | 2.24 | 52.01 | 0.00 | -0.32 | -0.16 |
|  | High | 1.62 | 2.14 |  |  |  |  |
| Positive image | Low | -0.52 | 2.23 | 310.06 | 0.00 | -1.07 | -0.47 |
|  | High | 1.77 | 2.03 |  |  |  |  |
| Condition | Low | 0.66 | 2.44 | 157.363 | 0.00 | -0.56 | -0.27 |
|  | High | 1.86 | 1.80 |  |  |  |  |

*-We are very grateful for the comment reviewer gave. If reviewer gives us additional comments, we will revise it again. Thanks again for very good comment-*
