# Supplementary material for: Why do people resist AI-based autonomous cars?: Analyzing the impact of the risk perception paradigm and conditional value on public acceptance of autonomous vehicles
Source: PLoS One. 2025 Feb 6;20(2):e0313143. doi: 10.1371/journal.pone.0313143 (PMC11801528; doi:10.1371/journal.pone.0313143)
Supplement: Attachment — Submitted filename: Revision Note-version 100.docx [file pone.0313143.s003.docx]

Cover Letter

20 September 2024

Dear Editors:

We submit an revisited manuscript entitled “Why Do People Resist AI-based Autonomous Cars?: Analyzing the Impact of the Risk Perception Paradigm and Conditional Value on Public Acceptance of Autonomous Vehicles” for consideration by PLOS ONE.

Comments from reviewers were very helpful for the development of the paper. We tried to revise all of things that the reviewers pointed out. Reviewers’ comments gave us very valuable insight and direction for revision.

I confirm that this study is original and is our own work. It has not been published elsewhere, nor is it currently under consideration for publication elsewhere.

It has been checked by a native English speaker with expertise in social sciences.

Given its research focus and methodology, I reckon that it is more appealing to a scientific audience.

Lastly, the authors are available to review at least three new submissions for PLOS ONE within the next 12 months.

I have no conflicts of interest to disclose.

Please address all correspondence concerning this manuscript to me at

seoyongkim@ajou.ac.kr.

Thank you for your consideration of this manuscript.

Sincerely,

Seoyong Kim

Comment from Reviewer 1 and Revision

□ Reviewer #1

□ Comment 1: Well done, I recommend slight Minor English proofreading, it is necessary now.

Well done, I recommend slight Minor English proofreading, it is necessary now.

○ Answer: We made the English proofreading.

*-We are very grateful for the comment reviewer gave. If reviewer gives us additional comments, we will revise it again. Thanks again for very good comment-*

Comment from Reviewer 2 and Revision

□ Reviewer #2:

□ Comment 1: Thanks for providing detailed responses to my comments and making the necessary changes. The manuscript looks in good shape now and I would recommend acceptance.

○ Answer: Thank you for giving the good review for us.

*-We are very grateful for the comment reviewer gave. If reviewer gives us additional comments, we will revise it again. Thanks again for very good comment-*
